# Ten quick tips for building FAIR workflows

**DOI:** 10.1371/journal.pcbi.1011369

**Published:** 2023-09-28

**Authors:** Casper de Visser, Lennart F. Johansson, Purva Kulkarni, Hailiang Mei, Pieter Neerincx, K. Joeri van der Velde, Péter Horvatovich, Alain J. van Gool, Morris A. Swertz, Peter A. C. ‘t Hoen, Anna Niehues

**Affiliations:** 1 Medical BioSciences Department, Radboud University Medical Center, Nijmegen, the Netherlands; 2 Genomics Coordination Center and Department of Genetics, University of Groningen, University Medical Center Groningen, Groningen, the Netherlands; 3 Translational Metabolic Laboratory, Department of Laboratory Medicine, Radboud University Medical Center, Nijmegen, the Netherlands; 4 Department of Human Genetics, Radboud University Medical Center, Nijmegen, the Netherlands; 5 Sequencing Analysis Support Core, Department of Biomedical Data Sciences, Leiden University Medical Center, Leiden, the Netherlands; 6 Department of Analytical Biochemistry, Groningen Research Institute of Pharmacy, University of Groningen, Groningen, the Netherlands; SIB Swiss Institute of Bioinformatics, SWITZERLAND

## Abstract

Research data is accumulating rapidly and with it the challenge of fully reproducible science. As a consequence, implementation of high-quality management of scientific data has become a global priority. The FAIR (Findable, Accesible, Interoperable and Reusable) principles provide practical guidelines for maximizing the value of research data; however, processing data using workflows—systematic executions of a series of computational tools—is equally important for good data management. The FAIR principles have recently been adapted to Research Software (FAIR4RS Principles) to promote the reproducibility and reusability of any type of research software. Here, we propose a set of 10 quick tips, drafted by experienced workflow developers that will help researchers to apply FAIR4RS principles to workflows. The tips have been arranged according to the FAIR acronym, clarifying the purpose of each tip with respect to the FAIR4RS principles. Altogether, these tips can be seen as practical guidelines for workflow developers who aim to contribute to more reproducible and sustainable computational science, aiming to positively impact the open science and FAIR community.

This is a *PLOS Computational Biology* Software paper.

## Introduction

Technological advancements in data-driven research disciplines come with larger data volumes and complexity. The significant increase in amounts of data has a negative impact on the already existing reproducibility crisis [1,2]. These developments call for the use of repeatable and reviewable workflows. Workflows are systemic executions of multiple computational methods to analyze datasets, thereby fitting solutions to gain meaningful insights from raw, heterogeneous data [3].

Previously, the FAIR (Findable, Accessible, Interoperable, Reusable) principles have been introduced to serve as guidelines for good scientific data management [4]. These principles have been adapted to the FAIR for Research Software (FAIR4RS) principles [5,6], which are designed to improve portability, reusability, and sustainability of research software. Thus, applying the FAIR4RS principles on research workflows (FAIR workflows) will enhance overall reproducibility and reuse in research, supporting maturation of the open science and FAIR communities. It is important to realize that these FAIR workflows are not to be confused with data FAIRification workflows, which can be used to make FAIR data. For the FAIRification of (research) data, we refer to the original FAIR guiding principles [4].

Practical guidelines have previously been described that reflect specific use cases implementing the FAIR/FAIR4RS principles. For example, recommendations have been introduced regarding research data discovery [7], reproducible computational research [8], and the interoperability of individual computational tools [9]. However—to the best of our knowledge—no guidelines are available that focus specifically on FAIR workflows.

**Fig 1:**
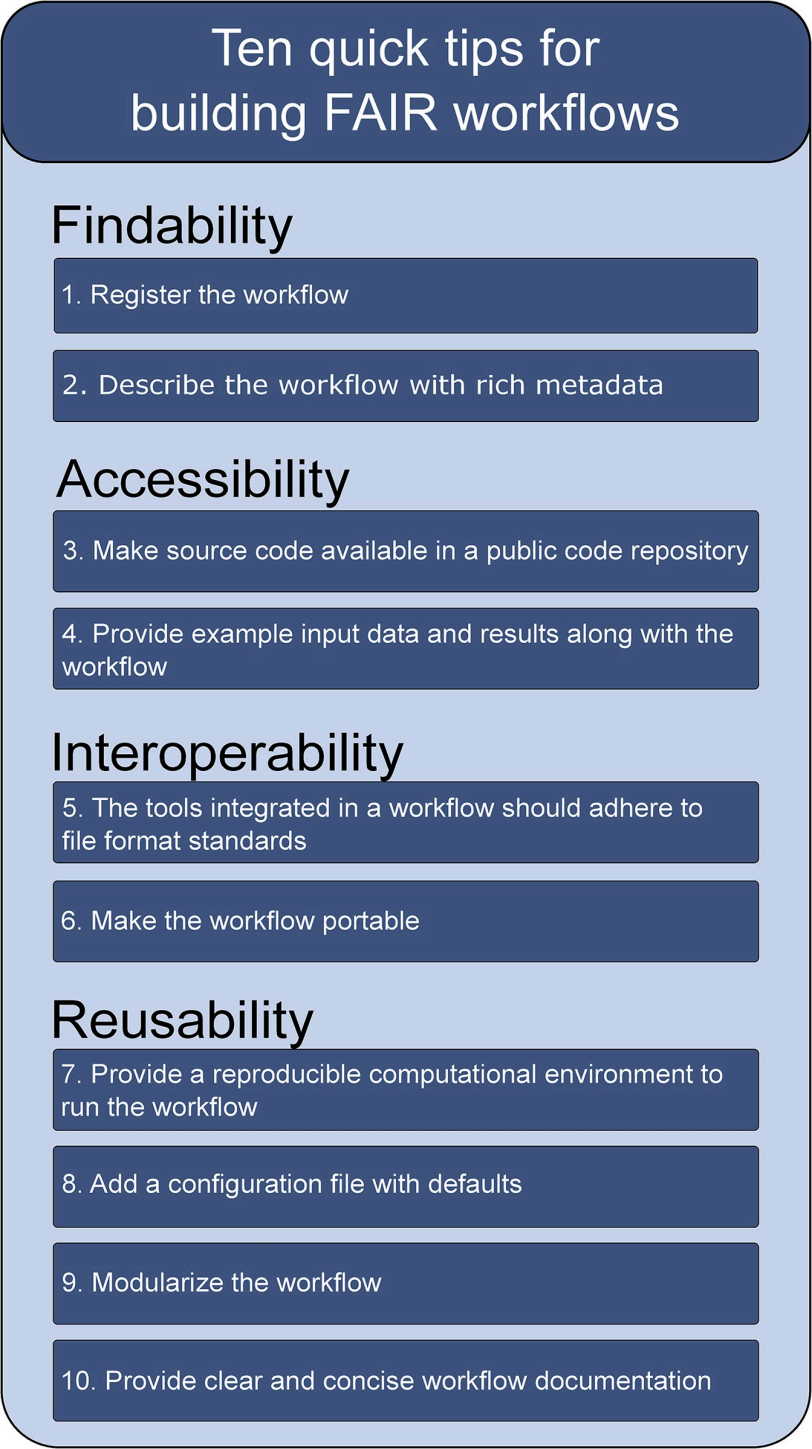
Ten quick tips for building FAIR workflows.

In this article, we propose 10 quick tips (Fig 1) for researchers working on computational workflows in any discipline. These tips should assist researchers in the development of FAIR workflows. The tips are following the FAIR4RS principles and have been developed and discussed with a group of experienced workflow developers in the context of the Netherlands X-omics initiative [10], a large-scale research infrastructure for the generation, analysis, integration, and stewardship of molecular omics data (genomics, proteomics, metabolomics, etc.). We aimed for a set of tips that are applicable to any computational environment and are relevant for any research discipline. However, we are aware that not every technical solution can be applied to every type of research workflow, as workflow designs can be highly diverse across different research fields. Hence, we present multiple technical solutions for each tip and encourage researchers to keep track of novel future workflow/software technologies that could further improve FAIR workflow development.

## Findability

### Tip 1: Register the workflow

FAIR workflow development starts by making it findable. Registering the workflow to any public record, preferably one that is also indexed by popular search engines, will increase findability. While general software repositories such as Maven and Dockerhub have value, ideally, we recommend registries that enable systematic scientific annotations and are catering for workflows written in different languages. Examples of these registries are WorkflowHub [11] and Dockstore [12].

WorkflowHub, sponsored by the European RI Cluster EOSC-Life [13] and the European Research Infrastructure ELIXIR [14], enables researchers to publish their workflows and thereupon can be discovered and reused by others. Currently, WorkflowHub supports multiple widely used workflow languages, such as the Common Workflow Language (CWL) [15], Snakemake [16], Nextflow [17], and Galaxy [18], through which many workflows can be collected into one place. WorkflowHub can assign a unique and persistent identifier—digital object identifier (DOI)—to the workflow, making it easily citable. Whenever a new workflow version is published, another DOI is automatically minted, making all workflow versions findable and citable. Workflow DOIs are registered with DataCite [19], and workflow metadata (see Tip 2) are automatically added to the knowledge graphs DataCite Commons PID Graph [20] and OpenAIRE Research Graph [21]. These can be used as platforms to explore research data, software, publications, etc., in a number of records.

Dockstore—made in collaboration with the Global Alliance for Genomics and Health (GA4GH) [22]—supports workflow languages CWL [15], Workflow Description Language (WDL) [23], Nextflow, and Galaxy. With Zenodo as DOI provider [24], unique identifiers can be minted for specific workflow snapshots (versions) in Dockstore.

On the other hand, there are workflow language-specific registries, for example, nf-core (which contains only curated Nextflow workflows) [25], Galaxy [18], the Snakemake workflow catalog [26], and KNIMEhub [27]. Although such workflow registries do not offer the possibility to generate DOIs, the workflow’s source code can be made citable with Zenodo by linking the respective code repository.

### Tip 2: Describe the workflow with rich metadata

Describing the workflow with rich metadata enables both humans and machines to understand what the workflow does and supports its findability by search engines. The metadata should cover information on all data entities that are present in the workflow, such as workflow language files, scripts, configuration files, example input data, as well as characterization of the purpose, scope, and limitation of the workflow to facilitate workflow discovery.

Most workflow languages that are mentioned in this paper enable researchers to add generic metadata (e.g., authors, organization, project title), which can already help end-users to understand the workflow. CWL offers the possibility to add domain-specific metadata (programming languages, file formats, tools used, versions, etc.) as well, so that all workflow elements are formally described with ontology terms—preferably from the EDAM ontology, which focuses on data analysis and management [28].

Furthermore, research data can be packaged along with the associated metadata using the RO-Crate (Research Object Crate) specification [29]. A workflow RO-Crate should follow the community curated Bioschemas [30] specification for a computational workflow, which defines the workflow properties that are mandatory or recommended to be described [31]. The metadata is captured in a JSON-LD file, using the Linked Data principles [32]. Following these principles, the metadata file describes all data and contextual entities (researchers, organizations, etc.) of the workflow with uniform resource identifiers (URIs). This ensures that all entities in the RO-Crate are described unambiguously and can be easily searched for. Moreover, workflow RO-Crate objects can be directly uploaded to WorkflowHub to register the workflow. Altogether, the RO-Crate method offers a good trade-off between usability (human readable formats) and richness (sufficient metadata).

An example how metadata aids discovery is showcased by Bio.tools [33,34]. a registry developed by ELIXIR, the European Infrastructure for Biological Information. Bio.tools has a broader scope not only registering workflows but also other tool artifacts such as databases and software tools. It is of particular interest for the community as it demonstrates how rich metadata has added value for discovering relevant workflows: Ontology annotations can be used to tag the workflow’s purpose and scope, thereby enhancing findability.

## Accessibility

### Tip 3: Make source code available in a public code repository

With the workflow’s source code available on a public code repository, anyone can access the software using commonly used communication protocols (HTTPS or SSH). Multiple conventional repository services for software development are available such as GitHub, GitLab, and Bitbucket, allowing code sharing via the Git protocol (which uses either HTTPS or SSH). Because the Git protocol to retrieve software is free of charge and implementable on any system [35], it is a recommended solution for making a workflow accessible.

Source code should be written following widely used style conventions, e.g., PEP 8 for Python [36] and the Google Style Guide for a variety of programming languages [37]. Code analysis tools that can assist workflow developers in adhering to these style conventions are available [38–41]. These tools can be integrated in the workflow development routine, for example, through automatic testing protocols (see Rule 4) that check if the code follows predefined style schemas. By doing so, this ensures that each modification will be written in readable and concise code.

Complete accessibility is more than providing the workflow’s source code on a code repository: The workflow’s source code should be accompanied with clear open source licensing. Licenses are meant to protect software owners and users, by specifying the permissions and limitations of the user, as well as the conditions that have to be met when reusing software. As there are many different open licences available, we can refer to choosealicense.com [42], which helps workflow developers to find a suitable license.

Code repositories offer other useful features. For example, version control using standardized protocols on the pipeline’s code will increase both findability and accessibility. As mentioned above, WorkflowHub and Dockstore provide an option to keep track of different workflow versions. Hence, combining the Git protocol and one of these workflow registries will enable the end-user to both find and access a specific version of the workflow. This will make it possible to reproduce any results generated by the workflow and not limit the end-user to the most recent version.

### Tip 4: Provide example input data and results along with the workflow

Accessibility of the workflow’s input data and associated results will help the end-user to understand how the workflow should function and improves reproducibility. Example data can be provided along with the workflow, for example, when using RO-Crate to package the workflow. Alternatively, the workflow documentation should give guidance on how to retrieve the data, preferably from a FAIR data repository. When the input data are privacy-sensitive, names/patient IDs should be pseudonymized to ensure privacy protection. If the data are too sensitive to be shared in any manner, synthetic data can be generated that mimics the original data, for example, by random sampling of the original data’s distribution.

Moreover, example data can be used to verify the users’ configuration. Running a pipeline in another computational environment can require adjustments to the configuration file (see Tip 8). The example data with results can be used to verify that the workflow runs correctly with this new configuration profile.

All workflow results are best collected through comprehensive rendered reports. Workflow managers like Nextflow and Snakemake support automated report generation. The readability and accessibility of these reports is particularly useful for non-computational researchers. The configuration profile that was used to run the workflow should be included in the report in order to properly document the parameters used and steps that were taken to produce the results.

Additionally, test functions can be incorporated in the workflow to guarantee a proper workflow execution and, if not executed correctly, reveal quickly where the execution halts. An interesting tool for workflow sustainability and reusability is the LifeMonitor project from EOSC-Life [13]. This service, available as both API and web application, facilitates automatic testing of workflows with given example data. Being interoperable with GitHub and WorkflowHub/RO-Crate, the LifeMonitor can be easily used for the FAIR workflow. Furthermore, whole workflow testing frameworks, such as *pytest-workflow* [43], enable researchers to write test configurations (YAML files) for any workflow type. These configurations check whether the workflow components produce the correct files (file paths, MD5 checksums, lists of strings present in the file) or exit according to the predefined command exit code.

Unit tests are small tests that can be implemented in a workflow to test the execution of single scripts or even functions within a script. For popular programming languages, there are libraries available that are designed to implement unit tests, for example, the built-in *unittest* library [44] for Python and *testthat* [45] for R. Notably, some of the current workflow managers are able to automatically generate unit tests (Snakemake). Others offer workflow language-specific testing frameworks (KNIME, Galaxy). Ideally, these tests are automated such that they continuously verify if latest versions of the workflow still function as expected, a practice known in software engineering as “Continuous Integration and Continuous Delivery (CI/CD)” [46]. CI refers to the automatic builds, tests, and integration of new code features, where CD means the automatic release of new software (versions), with the main developer’s approval [47]. Multiple public code repositories offer the possibility of automatic workflow testing via CI/CD. All things considered, automatic testing of the workflow’s source code can increase time efficiency of workflow development and secure workflow quality.

## Interoperability

### Tip 5: The tools integrated in a workflow should adhere to file format standards

Adopting standardized file formats increases interoperability. Not only workflow in- and output files, but also intermediate files that are exchanged by processes within the workflow should be written in standardized formats where possible. This facilitates the reuse of individual workflow components (see Tip 9) in other workflows.

Data format standards can be highly diverse. For workflows, we can distinguish between unprocessed input files and intermediate files. For example, regarding unprocessed input data in the bioinformatics field, many different file formats are considered to be standard. Nucleotide sequencing data are commonly saved as SAM files or derivatives (BAM, UBAM, CRAM, VCF) and metabolomics/proteomics as mzML or MAF files [48]. These formats are useful when data-specific tools are used within workflows, as these tools are written and optimized for these standards. For more general data analysis components of the workflow, it is recommended to use file formats that are commonly used to read and write data frames in popular programming languages like R and Python, such as CSV and TSV. For more complex data, file formats can be used that are more uncommon, but allow embedding of different data types, such as JSON, XML, or RDF.

Large datasets might require large amount of disk space, limiting reusability. In such instances, it is worth to consider compressed file formats that can be easily read in, for example, Python, R, and command line tools. Additionally, there are tools available [49,50] that allow researchers to read and write indexed compressed files, reducing memory consumption even further and enhancing data retrieval speed. Alternatively, there are binary compressed file formats, such as HDF5 [51] and ZARR [52]. These do not only offer high efficiency, but also facilitate interoperability, by enabling the annotation of column data types.

It is important to realize that current data standards might not be persistent over time. Using data standards does not mean being blind to emerging data standards that possibly offer more advantages. In the long run, it is a community effort to determine which domain-specific standards should be retained or replaced by better alternatives. Therefore, we recommend closely keeping track of the latest developments in the respective field that a researcher is working in. Resources are available to assist researchers in staying updated with the latest data standards, such as FAIRsharing [53] and the FAIR cookbook [54]. Meanwhile, using formats that also can be parsed as raw text files (as compared to proprietary binary formats) reduce the chance that data cannot be accessed.

### Tip 6: Make the workflow portable

By utilizing workflow managers, workflows can achieve higher portability, allowing them to operate seamlessly across different types of computational environments. Workflow managers are designed to streamline workflow development and can simplify the implementation of the technical solutions that increase portability (software containers, workflow configuration, workflow modularization, etc.).

It is recommended to use one of the actively used workflow managers that are portable, scalable, and have sufficient documentation, such as Nextflow, Snakemake, and Galaxy [55]. WorkflowHub is compatible with multiple workflow languages, which all have their pros and cons. An extensive review on workflow managers [55] provides a comprehensive analysis of workflow systems in various characteristics, including portability, reusability, ease of use, and scalability, among others. However, it is important to realize that workflow managers are evolving rapidly and that these reviews can become outdated quickly. Nonetheless, the workflow management system of choice depends on the specific use case, for which recommendations have previously been described [56].

Using one of the aforementioned workflow managers will strongly ease the development of a FAIR workflow. On the downside, while still usable through a command line interface, the workflow would not necessarily be interoperable with workflows written in different workflow languages or built with future workflow managers. However, some workflow languages can be run with different workflow engines. For example, workflows written in CWL can be run with Cromwell [57] and Galaxy, whereas workflows written in WDL can be run with Cromwell and miniWDL [58]. Alternatively, when using non-interoperable workflow managers, a CWL description on top of the workflow language can be added in order to achieve higher portability.

## Reusability

### Tip 7: Provide a reproducible computational environment to run the workflow

Irreproducible research results can be caused by small differences in computational environments, which can be simply differences in Python/R versions, library versions, or operating systems. Computational environments provide users with the capability to execute the entire workflow on the same system that was used by the workflow developers, requiring substantially less effort compared to installing all workflow dependencies from scratch. With this in mind, it is important that reproducible computational environments are provided for the end-user.

Available tools that are specifically designed for this include frameworks for building (scientific) software such as EasyBuild [59], which automates the building of software on HPC platforms. For Python and R, package managers Conda [60] and *renv* (only for R) [61] can be used to create computational environments with installations of specific library versions. These environments can be transferred as YML files, which define the library versions. Many workflow management systems can integrate *conda* and/or *renv* environments, through which these installations are facilitated.

Alternatively, software containers can be used. Software containers are lightweight computational environments containing all necessary elements (code, dependencies, data, configuration, etc.) to execute a certain process [62]. For example, a software container used for research can be a simple Linux environment containing only a specific Python version installed together with analysis packages such as *Tensorflow* [63].

A popular technology for software containers is Docker [64]. Docker container images are built following recipes—so-called Dockerfiles—which can be used as both human and machine-readable documentation of the container. Therefore, it is crucial to write understandable Dockerfiles [65]. Most Dockerfiles start with a parent image, on which new installations are made. In the previous example, the “simple Linux environment” can be a parent image, which is exemplary for the ease-of-use of Docker containers. Various workflow managers can directly pull software containers from container registries such as Docker Hub, so that researchers do not need to build the software containers manually. Note that inactive container images are not perpetually retained in services such as Docker Hub, which should be prevented with FAIR workflows. Docker containers are currently most frequently used, because of their ease-in-use, platform in-dependency, and the high number of base images available on Docker Hub.

Alternative container engines are Podman [66], Charliecloud [67], Shifter [68], and Apptainer—formerly known as Singularity [69]. These software container platforms are interoperable with Docker: it is possible to run Docker containers and pull base images from Docker registries with these engines.

### Tip 8: Add a configuration file with defaults

Possibility to parameterize the workflow to different use cases greatly enhances reusability. In our experience, we prefer parameterization using config(uration) files over other forms of parameterization (such as command-line parameters) because the files themselves improve FAIRness of the workflow use.

A config file can be used to fine-tune the workflow execution on different levels (software/hardware), through which it can be run in different computational environments without the need to modify the workflow implementation. For example, file paths of both input and output data can be specified in the configuration file. To put it another way: hardcoded paths/settings should never be present in a research workflow, but stored in the configuration file. Moreover, storing all intermediate results may require large storage space, more than is required to store input and output files together. Workflows should therefore provide configuration options to manage intermediate files (i.e., keep or delete it) to avoid data explosion during workflow execution. Equally important for workflow reproducibility are hardware specifications in the config file. When large amounts of data are to be processed, hardware settings such as GPU/CPU numbers and RAM amounts can be specified to scale the workflow execution to the respective computing environment.

As discussed in Tip 7, different tools are available to provide reproducible computational environments. Ideally, the end-user can select any preferred tool to build such an environment, which could be specified in the config file as well. A good example are the curated nf-core pipelines, which can be run with several software container engines (Docker, Apptainer, Podman, Shifter, and Charliecloud) and Anaconda. Evidently, this increases both workflow interoperability and reusability. To simplify the development of workflows that can be executed with both Conda and containers, researchers might consider Snakemake’s capability to containerize a workflow originally built using pure conda environments.

We encourage workflow developers to add default values in the config file, saving time and effort for the use cases that do not require extra workflow customization. For the use cases that need specific workflow configurations, sufficient documentation on both the workflow and the configuration options should be provided along with the workflow (see Tip 10). Workflow managers mostly include their own config files, making it easier for both workflow developers and end-users to implement configuration files.

### Tip 9: Modularize the workflow

To enhance reusability, we recommend building workflows in a modular structure.

Firstly, alternative workflow designs are facilitated with modular workflows. If researchers are interested in reusing only a specific part of the workflow, this part can be easily imported. If a specific software container is assigned to every module—these can be part of the import—higher workflow reproducibility is obtained. Good examples of readily deployable workflow modules are the nf-core-modules and snakemake-wrappers (and meta-wrappers).

Secondly, modular workflows are simpler to understand and can be maintained more efficiently. For example, when creating a code repository that contains all the reusable modules, which are imported by other workflows, only this module repository needs to be in active development. Besides saving time and effort, this ensures that the exact same modules are reused in all different workflows. And logically, simpler maintenance will result in less code writing and thus fewer bugs.

Another approach for workflow modularization is creating a software package that can be used outside of the workflow. Although this requires additional effort, it opens up opportunities for uses other than just running or adapting the workflow.

### Tip 10: Provide clear and concise workflow documentation

Since end-users initially get familiar with the workflow through its documentation, it is essential for reusability. Documentation can be provided in multiple forms: code repositories (README files), workflow registries (HTML web page), or workflows themselves (––help parameter) can all be included in the user documentation. We recommend adding documentation in as many forms as possible, while preserving uniformity to prevent any confusion for users. Therefore, it is advisable to use one as leading documentation, which is to be transformed into the other documentations, saving time and effort as well. Alternatively, different workflow documentation sources can link to another. For example, the workflow’s help parameter provides the most basic information on workflow usage to end-users and links to the workflow registry page with more extensive documentation.

In addition, we recommend equipping the documentation with a flowchart that gives a schematic overview of the different workflow components and how these are connected. On top of that, a text document/table can provide more detailed information. This would include every workflow process, script, input/output files, and workflow parameters used. With every pipeline step documented, reuse and re-implementation of the workflow is made easier.

Finally, the source code of the workflow can also function as documentation. As highlighted in Tip 3, source code should be written following widely adopted style conventions in order to make the code more readable. With the source code being more self-explanatory, readers can easily discover the purpose of each code segment, thereby understanding how the workflow operates on a more detailed level. Also, writing the source code in a modular fashion can greatly enhance its overall readability.

## Conclusion

Increasing data volumes and complexity in research are both an opportunity and a challenge that require more creative and resourceful workflow designs. To structure this diverse landscape of different workflows, the FAIR4RS principles can play a significant role. Here, we have introduced a set of 10 quick tips that can help to navigate through these principles when developing a scientific computational workflow, irrespective of the research field. For each tip, we propose multiple technical implementations that can be used, because we are aware that not every technical solution can be applied to every workflow at any time. Inevitably, future technological developments will lead to additional useful tools for workflow FAIRification. We believe in the added value of these tips to build a stronger and sustainable workflow community, where reusable, trustworthy, and validated workflows are the standard in any data-driven research field.
